# Implicit Knowledge Acquisition and Potential Challenges for Advanced Chinese and Spanish EFL Learners: A Word Monitoring Test on English Questions

**DOI:** 10.3390/bs13020099

**Published:** 2023-01-24

**Authors:** Qiaoling He, Isabel Oltra-Massuet

**Affiliations:** 1 College of General Education, Sichuan International Studies University, Chongqing 400031, China; 2 Department of English and German Studies, Universitat Rovira I Virgili, 43002 Tarragona, Spain

**Keywords:** implicit knowledge, English questions, morphosyntactic inflections, grammatical sensitivity, EFL learners

## Abstract

This study aims to explore whether advanced EFL learners can acquire implicit knowledge of basic sentence structures, such as English questions. We ran a reaction-time experiment, a word monitoring test experiment to test learners’ implicit knowledge by checking advanced EFL learners’ grammatical sensitivity to English questions with five types of grammatical errors. The study recruited three groups of participants: native English speakers (*n =* 12), advanced Chinese EFL learners (*n =* 32), and advanced Spanish EFL learners (*n =* 37). Our results revealed that advanced EFL learners had not yet attained native-equivalent implicit grammar knowledge in English questions, despite their English proficiency level. The results also indicated that the learners’ different L1 languages do not impact advanced learners’ overall implicit knowledge acquisition but constitute influential factors for particular morphosyntactic inflections in English question formation.

## 1. Introduction

One important topic in second language acquisition (SLA) concerns L2 learners’ acquisition of implicit knowledge and, specifically, to what extent L2 learners can acquire implicit language knowledge. A high level of implicit knowledge correlates with learners’ high linguistic competence [1,2,3]. Therefore, one would assume that advanced language learners should have acquired a high degree of implicit knowledge. Studies regarding implicit language knowledge have mainly concentrated on distinguishing implicit from explicit knowledge [3,4,5,6,7,8] and developing and validating research tools for measuring the two kinds of knowledge [1,5,9,10,11,12,13,14,15,16]. To push forward and generalize previous findings on this topic, further studies on L2 learners with different L1–L2 combinations and on other linguistic structures are needed [11,17]. This study measured the implicit knowledge levels of a native English-speaking group and two groups of advanced EFL learners from different L1 backgrounds (Mandarin Chinese, hereafter referred to as Chinese, and Peninsular Spanish, hereafter referred to as Spanish), using the word monitoring test (WMT) experimental paradigm. We also conducted a comparison of the implicit knowledge levels between native speakers and the two advanced EFL learner groups. The present study seeks to contribute to the debate on whether advanced EFL learners can acquire implicit knowledge of English questions that are equivalent to the native speakers’ level and whether English learners from different L1s encounter specific difficulties in the acquisition of particular kinds of morphosyntactic features in English questions.

To address the aforementioned research concerns, we raised the following two research questions for the present study.

**RQ1:** Did advanced EFL learners acquire implicit knowledge that was equivalent to the level of native speakers?

**RQ2:** What might potentially impact Spanish and Chinese EFL learners’ implicit knowledge acquisition, in terms of English question formation?

## 2. Literature Review

### 2.1. Acquisition of English Questions for Chinese and Spanish EFL Learners

The acquisition of morphosyntactic inflections in question formation is difficult for L2 English learners since the syntactic manipulation of interrogatives is far more complex than the syntax of statements [18]. Diversified errors do appear when building English questions for both preliminary Chinese and Spanish EFL learners [19]. One reason for this difficulty may come from the syntactic differences between L1 and L2 question formation. Chinese is a so-called “*wh* in situ” language [20]. That is, in Chinese, the word order is stable, and there is no *wh*-movement, nor any subject-verb inversion or auxiliary insertion in questions. Chinese yes/no-questions are formed by adding particles, such as *ma* or *ba*, at the end of a declarative sentence (as shown in (1c)), with no morphological inflection on verbs [21]. Meanwhile, Spanish, despite belonging to the same Indo-European language family as English, significantly differs from English. In Spanish, verbs inflect for tense, aspect, and mood, and show morphosyntactic agreement features (person and number) in all forms, so that pronoun subjects are generally left unexpressed. Simplifying things somewhat, while Spanish *wh*-questions do show *wh*-movement, as in English, but without auxiliary *do*-support or subject–auxiliary inversion, basic Spanish yes/no questions are built by just changing intonation in sentences, as exemplified in (1b) and (2b). Although Spanish does have auxiliaries, such as *haber*, “have”, or *ser*, “be”, for perfective and passive tenses, respectively, their syntactic behavior differs from those in English [22].

To illustrate the syntactic differences between English and Spanish auxiliaries, see the contrast in (i)–(ii), where examples can be said to be word-by-word translations.

(i) English: John has gone to Tarragona.—Has John gone to Tarragona? vs. *John has gone to Tarragona?
(ii) Spanish: Juan ha ido a Tarragona.—*Ha Juan ido a Tarragona? vs. Juan ha ido a Tarragona?

The following example questions in English, Chinese, and Spanish illustrate basic morphosyntactic differences in question structures in these three languages. We use the following abbreviations for glosses: 2: second person; 3: third person; sg: singular; pl: Plural; PrsInd: Present Indicative; q: Question marker.
(1) *Yes-no* Questionsa. English:Doyou get permission to enter the office?
b. Spanish:¿Tienespermisoparaentrara laoficina?

get.2sg.PrsIndpermissiontoenterto theoffice?
c. Chinese:Nidedaoxukequjinbangongshi ma?
Yougetpermissiontoenterofficeq(2) *Wh*-Questionsa. English:Wheredotheywant tobuildtheirnewfactory?b. Spanish:¿Dóndequierenconstruirsunuevafábrica?
wherewant.3pl.PrsIndbuildtheirnewfactory?c. Chinese:Tamenxiangzainalixiujiantamen dexingongchang?
Theywant.toatwherebuildtheirnewfactory?

Morphological inflection is problematic for FL/L2 learners from various L1 [23] languages, as it is relatively easy to study inflection but it is difficult to acquire [24]. Studies on the acquisition of English questions for learners from different L1s (e.g., Chinese, Korean, Portuguese, and Spanish) revealed different morphosyntactic errors in forming English questions from different perspectives. Cuza (2016) [25] reported that Spanish–English bilingual learners showed a low level of inversion in forming *wh*-questions and embedded questions [25]. In a corpus study of learner errors, McCauley et al. (2019) [26] reported a high frequency of non-inversion errors in children’s spontaneous production of *wh*-questions [25,26]. Pozzan and Valian (2017) [27] found that Spanish and Chinese learners displayed comparable subject–verb inversion errors when forming English questions [27]. Ma (2018) [28] found a high occurrence of inversion errors in the production and judgment of English embedded *wh*-questions by Cantonese learners of English. More importantly, those learners who seem to have gained a high communicative ability still produce basic English grammatical errors, such as dropping plural markings or creating third-person present-tense morphemes. Acquisition difficulty in morphology is also reflected in morphosyntactic inflections, in the context of forming English questions. For example, Hong Kong ESL learners had difficulty in acquiring *wh*-questions, and exhibited grammatical errors when using *be* and *do* (e.g., *What is Miss Wong say?), or failing to invert verb structures [29]. Moreover, Zhu and Wu found that intermediate and advanced Chinese learners’ interpretation of the discourse function of three different types of *yes/no* questions in their L1 language affected their choice of linguistic forms [21].

Although a series of studies have investigated the difficulties found in acquiring morphosyntactic features in English questions from different perspectives, morphosyntactic errors in English question formation have not been systematically reviewed and categorized. To find out whether cross-linguistic transfer from L1 hindered the formation of English questions in preliminary L2 learners, He and Oltra-Massuet [19] classified five types of errors, including GAUXC errors (erroneous use/wrong choice of the auxiliary verb, e.g., *Is your son like doing extreme sports as much as you?), GAUXT errors (mistakes involving tense and its related morphology, e.g., *Before their final decision, what do they say in the meeting?), GAUXA errors (errors in the agreement between the subject and the verb, e.g., *Does they agree to sign the contract in seven days?), GAUXO errors (errors in word order between the auxiliary and content verb, e.g., *Shall be we working for that company for a whole year?), and GAUXM errors (mistakes involving the morphology of the verb, e.g., *Have your students conduct the experiment all by themselves?). Among all five categories of errors, grammatical errors made by Chinese preliminary learners were mainly distributed between GAUXC and GAUXT errors, while those from preliminary Spanish learners were evenly located among GAUXA, GAUXC, and GAUXT errors [19]. However, whether these morphosyntactic errors disappear or persist in advanced Chinese and Spanish learners remained unclear.

Therefore, a study targeting advanced EFL learners’ grammatical sensitivity to the fine-grained types of morphosyntactic errors displayed by preliminary learners was conducted to uncover the learners’ degrees of implicit knowledge acquisition. Moreover, focusing on a specific sentence structure with a group of advanced EFL learners from two different L1 backgrounds should reinforce the results regarding the potential influence of L1 on the learners’ acquisition of implicit knowledge.

### 2.2. Implicit Knowledge and Its Acquisition Issues

In the field of second-language acquisition (SLA), the concept of implicit knowledge has been defined in comparison with explicit knowledge, as its characteristics are elucidated, to a large extent, by the contrasting properties between the dyads of implicit and explicit knowledge. Ellis (2005) [30] differentiated implicit knowledge from explicit knowledge by identifying its seven key characteristics. That is, implicit knowledge is intuitive, procedural, and variable but systematic, automatic, non-verbalizable, and unlearnable [30]. Paradis (2009) [31] defined implicit knowledge in L2 as knowledge that individuals are not aware of, in terms of its grammar rule, but that can be inferred from their systematic behavior, allowing them to speak in a consistent way [31]. Ellis and Roever (2018) [11] further clearly defined implicit language knowledge as non-verbalizable knowledge that learners are not subjectively aware of but that they can access in spontaneous language production, via automatic processing [11].

Implicit knowledge is acquired in a natural and simple process that involves no conscious operations [4]. Although the definition of implicit knowledge has been evolving over time, there is a wide consensus in SLA that learners’ linguistic competence primarily consists of implicit knowledge, and that L2 production relies on implicit knowledge [7]. Importantly, it is broadly agreed upon that the ultimate goal of acquiring an L2 is to develop implicit knowledge [8]. However, to what extent L2 learners can acquire implicit L2 knowledge remains a matter of dispute, as there is a series of key variables involved that are not yet fully understood, such as L2 learners’ age-related issues [9] or learners’ L1 [10] or L2 residential experiences [11,12]. Therefore, it is important to study whether advanced EFL learners can acquire native-like implicit L2 knowledge and what might constitute the appropriate challenges by which L2 learners can acquire such knowledge. Theoretical linguists, from either the innatists’ or connectionists’ stances, showed a common grounding regarding the importance of implicit L2 knowledge in L2 learning, pursuing the same goal of elucidating whether implicit knowledge can be acquired and in what way L2 learners build up their implicit knowledge [3]; implicit knowledge, to a large extent, determines the L2 learners’ final attainment of language competence [3,16,18].

However, previous studies have shown differing views on whether L2 learners can acquire implicit L2 knowledge and on how implicit knowledge is acquired. In reflecting on frequency effects in language processing, N. Ellis (2002) [32] evaluated the roles of attention and form-focused instruction and summarized that form-focused instruction (FFI) can aid the acquisition of implicit knowledge. R. Ellis (2002) [33] reviewed 11 studies on the role played by FFI in implicit knowledge development, and supported the view of N. Ellis (2002) [32], suggesting that FFI can promote the acquisition of implicit knowledge. In studying whether L2 learners from diversified L1s acquired implicit lexical knowledge after the treatment of lexical form recall, recognition, and priming. Sonbul and Schmitt (2013) [34] found that these learners did not show improvement in the acquisition of implicit knowledge. However, by replicating the experiments of Sonbul and Schmitt (2013) [34], Toomer and Elgort (2019) [35] extended the findings of Sonbul and Schmitt (2013) [34] and found a certain degree of development of implicit knowledge in their participants.

By comparing cross-linguistic experiments, Hopp (2010) [36] showed that it is possible for adult learners with L1 English, Dutch, and Russian to achieve native-like attainment in terms of acquiring L2 inflections in German [36]. In exploring whether L2 learners acquired implicit knowledge of causative sentences with the verbs “have” and “get”, Zereshki and Rezaie (2018) [37] found that consciousness-raising tasks worked effectively on facilitating EFL learners in acquiring implicit knowledge of causative grammatical structure [37]. In examining whether Chinese learners of English can acquire knowledge of the past tense and past participles, Goad and White (2006) [38] showed that knowledge of both past tenses and participles in English is equally acquirable for Chinese speakers [38]. In studying the acquisition of L2 grammatical morphemes, Jiang (2011) [39] found that it is extremely difficult for Japanese learners to acquire English plural markers, although their acquisition is supposedly possible.

Existing studies [2,3,16,40,41,42] stated that learners’ language proficiency is revealed in their implicit knowledge. Advanced EFL learners, or high-proficiency learners, are learners with high language competence in terms of comprehension and production. It is, thus, reasonable to hypothesize that they possess a high degree of implicit knowledge. Therefore, measuring advanced EFL learners’ implicit knowledge, which is further checked against native speakers’ implicit knowledge, should allow the researchers to explore to what extent learners in an EFL context can acquire another language.

### 2.3. L1-Influenced Issues and The Morphological Congruency Hypothesis

As for whether EFL learners’ L1 influences their L2 morphosyntactic acquisition, previous studies [39,43,44,45,46,47] show diverse views. While they agreed that a learner’s L1 influenced their L2 acquisition, even if to different degrees, previous studies have offered various opinions on the role of such L1 influence. Some studies have held the view that L1 influence constantly interferes with L2 learners’ language acquisition [39,45], while others considered that L1 influence can be drastically reduced or even eliminated so that it does not substantially impact advanced learners’ acquisition of target structures as the learners’ proficiency increases [43,44].

In the study of the acquisition of plural noun phrases by Spanish and Korean learners of English, Ionin and Montrul [44] found that Spanish learners of English tended to transfer their L1 interpretation of articles with definite plurals to the target language, although the L1 transfer receded with the learners’ improved proficiency and L2 immersion, an effect that was especially visible in high-proficiency learners. Goad and White [38] found that the inflectional morphology of English tenses and past participles are both acquirable by Mandarin learners of English despite the absence of such inflections in their L1. Elston-Güttler et al. [43] also found that the L1’s influence in word processing had been expected and detected, but its influence decreased in high-proficiency learners when a semantic context was given. Pozzan and Quirk [48] suggested that the L2 English linguistic features predicted L2 learners’ accuracy in question formations related to word order, while both Chinese and Spanish learners’ L1 played a minor role.

Other research has suggested that the L1’s influence constantly affects L2 learners’ acquisition of target structures. These studies revealed that learners’ L1 had an influence on L2 morphosyntactic acquisition, and L1–L2 morphosyntactic congruity was one of the most prominent factors. Jarvis [45] found that the participants’ L1 constantly and detectably influenced their acquisition of content words. In studying the Spanish imperfect tense acquired by English learners of Spanish, Domínguez et al. (2017) [49] found that the L1’s influence on English features impacted the L2 learners’ acquisition of the morphology of the Spanish imperfective aspect. By comparing Russian and Japanese L1 learners of English, Jiang et al. [39] concluded that whether there is morphological congruency between L1 and L2 determines the L2 learners’ final attainment of morpheme acquisition. They stated that it is easier for learners to acquire a grammatical morpheme that is represented in their own L1. Furthermore, Jiang et al. [39] encouraged further studies exploring whether Japanese or Chinese learners of English can acquire native-like implicit knowledge of English morphemes that are not morphologically congruent in their L1. Gudmestad and Edmonds [50] found that the L1’s influence affected L2 learners’ acquisition of gender marking in Spanish and also encouraged more studies on different L2s with other L1 backgrounds to confirm the generalizability of the findings on L1 influence. Whether L1 influences advanced L2 learners’ acquisition of inflectional morphology is thus controversial and needs further exploration since studies on the acquisition of morphosyntactic inflection remain insufficiently explored. The present study seeks to contribute to this debate by focusing on advanced EFL learners from two different L1 backgrounds, to explore whether their L1 morphological features play a role in language acquisition for advanced learners and to investigate what morphosyntactic features in English questions remain difficult for advanced EFL learners, together with the influencing factors potentially impeding their acquisition.

The present study intends to explore this issue from the perspective of the morphological congruency hypothesis of Jiang et al. [39]. This was developed to delve into the issue of the “acquirability” of grammatical morphemes, especially from the perspective of the learners’ L1 influence on the L2 acquisition of particular morphological features, which helps to explain the difficulty and final attainment of L2 morphological features. This hypothesis is also in line with the feature-assembly hypothesis proposed by Lardiere (2009) [51]. Lardiere analyzed the comparison of the L1–L2 language system from the perspective of generative grammar and concluded that learners need to reset the existing L1 parameters or reassign the values to L2; if they fail to do so, they cannot attain L2 proficiency [51]. According to Jiang et al. [39], L1 and L2 are congruent in morphology when the meaning of a specific grammatical morpheme is grammaticalized and morphologically marked in both L1 and L2, whereas they are incongruent in morphology when there is no similar morphological marking for grammatical meaning in both L1 and L2.

Jiang et al.’s *morphological congruency hypothesis* is as follows:

“When L2 learners reach an advanced or near-native level of L2 proficiency, only congruent learners (i.e., those whose L1 has a corresponding morpheme to the target L2 morpheme) are able to reach native-like proficiency in acquiring an L2 morpheme. Incongruent L2 learners will find it extremely difficult, if not impossible, to develop native-like competence with respect to the same L2 morpheme.” (Jiang et al., 2011: p. 943) [39]

As illustrated by Jiang et al. (pp. 943–944) [39], their hypothesis was supported by previous research in three lines, which are:

(i) Advanced L2 learners encountered great difficulty in acquiring native-like knowledge in grammatical morphemes when the morpheme in their L1 was not congruent with the target morpheme in an L2.
(ii) For L2 learners with different L1s, learners with congruent L1 morphemes performed better than those without congruent L1 morphemes.
(iii) For L2 learners from the same L1 background who were learning different morphemes, the learners performed better concerning grammatical morphemes that were congruent than on those that were incongruent with their L1.

When exploring the potential challenges in the acquisition of English questions for the EFL learners under investigation, we formed three predictions, based on the morphological congruency hypothesis.

Prediction 1: Advanced EFL learners are confronted by great difficulty in the acquisition of native-like implicit knowledge of morphosyntactic features in English question formation that are not instantiated in their L1.

Prediction 2: Spanish EFL learners outperform Chinese EFL learners in acquiring morphosyntactic features in English question formation because there are morphological inflections on verbs in Spanish, but not in Chinese.

Prediction 3: For Spanish EFL learners, there are congruent morphosyntactic features and incongruent morphosyntactic features in question formation. Spanish EFL learners are expected to perform better on English morphosyntactic features that are congruent with morphological inflections in Spanish.

### 2.4. Hypotheses

The goal of the present study is to find out to what extent advanced EFL learners can attain implicit language knowledge and investigate whether EFL learners from two different L1s displayed a difference in their acquisition of implicit knowledge. This study further explores what might constitute difficulties for advanced EFL learners in acquiring implicit knowledge of morphosyntactic features in English question formation. Based on previous studies on the acquisition of implicit knowledge and the theoretical framework of morphological congruency, we formulated the following two hypotheses:

**Hypothesis 1** **(H1).** *Advanced EFL learners cannot acquire an implicit knowledge level equivalent to native speakers’ level*.

**Hypothesis 2** **(H2).** *Particular types of morphosyntactic inflections in English question formation constitute an influencing factor for EFL learners’ implicit knowledge acquisition of English questions.*

## 3. Materials and Methods

### 3.1. Participants

The study recruited 81 participants online, comprising 12 monolingual native English speakers and 69 advanced EFL learners, ranging from 20 to 40 years old. All advanced English learners were reported to possess a C1 English proficiency level (common European framework of reference for language (CEFR)), which was further confirmed via a short online C1-level test (Cambridge English test) immediately prior to completing the experimental tasks. The EFL learners consisted of two groups of participants: advanced Chinese EFL learners (*n =* 32) and advanced Spanish EFL learners (*n =* 37). We collected data from 75 participants, and obtained valid data from 69 participants, with an effective rate of 92%. Table 1 shows the participants’ basic demographic information.

### 3.2. Procedures

The experimental procedures in the present study were reviewed and approved by the Research–Innovation Ethics Committee from the Universitat Rovira i Virgili, Spain. The experiment was conducted online on the behavioral experiment platform, https://app.gorilla.sc/ (accessed on 25 July2021). All participants were recruited via the experiment participants’ recruitment platform, https://www.prolific.co/ (accessed between 20 June 2021 and 22 December 2021), which routes participants directly to the experiment platform. Every participant was required to give informed consent and read the experiment instruction before entering the experiment page. Participants started the experiment after filling out the background information questionnaire and passing the English proficiency test by presenting at least three correct answers out of five questions. The whole experiment lasted for about 30 min.

### 3.3. Experimental Instrument

#### 3.3.1. Word Monitoring Task (WMT)

There is a battery of tests that were designed by R. Ellis [3,30] to measure implicit knowledge, which includes the imitated oral elicitation test, the oral narrative test, and the timed grammar judgment, which have been employed widely in previous studies [1,16,30,52]. However, recent studies on the measurement of implicit knowledge [14,15,17] have suggested that tasks such as timed grammar judgment require participants to pay attention to linguistic forms and, thus, raise their awareness of related linguistic knowledge [53]. Suzuki and Dekeyser [15] found that the reaction time (RT) experiment, such as the word monitoring test (WMT), is a more effective tool to measure implicit knowledge as participants’ reactions are intuitive responses that are given within a limited time frame, while their focus is on the sentence’s meaning. The WMT experiment was designed based on the work of Jiang et al. [39] and Suzuki [14] and was modified for the present study based on the characteristics of experimental sentences, with questions instead of statements. The rationale underlying the WMT test is that if L2 learners have acquired a particular linguistic structure at an equivalent level to their native counterpart, they are supposed to be as sensitive as native speakers in displaying a similar degree of response delay regarding a violation of grammaticality in the linguistic structure under investigation.

**Figure 1:**
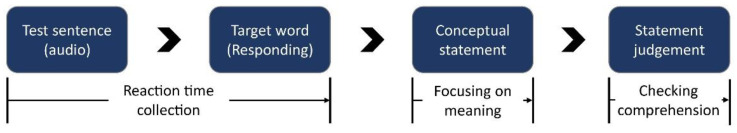
Experimental design.

**Figure 2:**
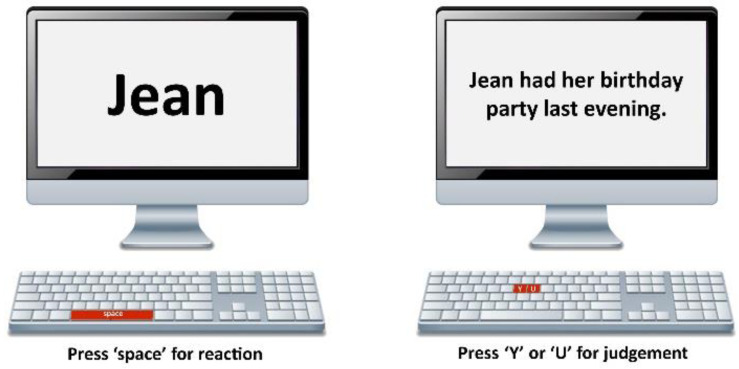
Illustration for the experimental operation.

The experimental procedure is displayed in Figure 1. In the experiment, participants were guided to listen to the audio recording of a sentence for a target word shown on the screen, then they needed to press a designated key as soon as they heard the target word (Figure 2). The morphosyntactic feature violation was designed to immediately precede the target word(s) in each critical experimental item. The target word was displayed on the screen before the response key was pressed, and participants were instructed to press the key immediately after they heard the target word. The reaction time that is recorded corresponds to the duration of the onset of the audio until the key-pressing action upon the appearance of the target word. A conceptual statement for checking the participants’ comprehension of the sentence was shown on the screen, and participants were instructed to press the corresponding key representing their judgment regarding the meaning. Thus, participants were directed to focus their attention on sentence meaning while identifying the target word. There was a practice session for participants to familiarize themselves with the experimental operation before they started the experiment.

An example of an experimental item is shown in the following:

Sentence audio: *Did she enjoyed Jean’s birthday party last evening?

Target word: Jean

Conceptual sentence for judging the meaning: Jean had her birthday party last evening (the Y key refers to the correct answer; the U key refers to the incorrect answer (see Figure 2).

While the participant was listening to the experimental sentence *Did she enjoyed Jean’s birthday party last evening? the target word *Jean* was shown on the screen. The participant should press the response key as soon as he/she heard the word “Jean”. The participant may or may not display a delay in responding to the grammatical violation of the verb enjoyed in *Did she enjoyed Jean’s…?, which immediately precedes the target word(s).

#### 3.3.2. Experimental Items

There were 5 practice items, 60 critical experimental sentences, and 30 filler items in the experimental design. Participants had to complete all 90 items in the experiment. Among the 60 critical experimental sentences, 30 ungrammatical sentences were experimental items, and 30 grammatical sentences were used as control items to calculate the RT. The grammatical sentences were used as “a baseline for determining if the phenomenon of lags in response, driven by implicit knowledge, is present” [54]. Thirty filler items were used to reduce the chances of the participant noticing the research focus of the stimuli items, that is, grammatical errors in question formation. The 30 fillers were all statements intended to conceal the fact that the experimental sentences were questions. Five types of grammatical errors were incorporated into the ungrammatical sentences. They referred to the findings regarding EFL learners’ grammatical errors in English questions reported by He and Oltra-Massuet [19]. The five types of grammatical errors are the choice of auxiliary (GAUXC), order of auxiliary (GAUXO), tense of auxiliary (GAUXT), auxiliary/verb morphology (GAUXM), and subject–verb agreement (GAUXA). All error types were incorporated as grammar violation features in the experimental items. Each type of error appeared twice in each list; thus, there are 6 items for each grammatical error type in total, with an equivalent of 6 grammatical sentences. All 60 experimental sentences were divided into three lists of experimental items, with one list consisting of 10 pairs of grammatical and ungrammatical items representing all five grammatical error types. The sequence of all experimental items, including grammatical, ungrammatical, and filler items, was randomly ordered so that participants could not predict the oncoming item. Examples of the ungrammatical and grammatical item pairs for each type of error are illustrated below.

Experimental items (five types, both grammatical and ungrammatical):

a. GAUXC: *Are you get the permission to enter the office?
  Do you get the permission to enter the office?
b. GAUXT: * Before their final decision, what do they say in the meeting?
  Before their final decision, what did they say in the meeting?
c. GAUXA: *Do your uncle live in the new neighborhood nearby?
  Does your uncle live in the new neighborhood nearby?
d. GAUXO: *Will be he giving a presentation at the conference?
  Will he be giving a presentation at the conference?
e. GAUXM: *Have you get a new job offer after the interviews?
  Have you got a new job offer after the interviews?

#### 3.3.3. Variable Manipulation

This study is designed to find out whether EFL learners can acquire implicit knowledge of typical English questions, as measured by participants’ reaction times in response to the target word in both grammatical and ungrammatical sentences. The reaction time reflects the participants’ sensitivity to grammar violations in morphosyntactic errors in English questions. For example, in the paired test sentences, *When did they went to the fun park yesterday?/When did they go to the fun park yesterday?, by comparing the participants’ Reaction Time (RT) on the target words *the fun park*, we can determine whether participants are sensitive to grammatical violations. The preposition “to” was not included as part of the target words, because of its status as an unstressed functional word, which is easily missed in audio recordings. As pointed out by Jiang (2013: p. 196), “One can be quite flexible in choosing targets. It can be any word that happens to be at a particular location in a sentence.” The concept of the grammatical sensitivity index (GSI) developed by Suzuki [55] was introduced in this study, comparing participants’ reaction times in response to ungrammatical and grammatical sentences. A longer delay in RT for the ungrammatical item revealed the participants’ grammatical sensitivity.

Each learner’s language background is an important variable that may influence their acquisition of implicit knowledge. Therefore, language background was manipulated as an independent variable in the study, which included Spanish and Chinese EFL learners as experimental groups and native speakers as the referring group. In addition, in order to detect what constitutes an influential factor in EFL learners’ implicit knowledge acquisition, we also introduced five types of morphosyntactic errors in question formation as an independent variable to measure their degree of acquisition, compared to native speakers’ data.

### 3.4. Statistical Analysis

After the experiments were completed, we pre-processed the data, and then input them into the Microsoft Excel database. The IBM Statistical Package for Social Sciences (SPSS) version 22.0 (SPSS Inc., Chicago, IL, USA) was used for statistical description and statistical inference. The participants’ demographic characteristics were described using descriptive statistics, including the frequency and constituent ratio. The difference in categorical variables in the demographics between groups was tested using the chi-square test, while the difference in the number of years of taking English classes between Chinese speakers (CS) and Spanish speakers (SS) was tested using an independent two-sample *t*-test, with the number of years of English classes as an independent variable and the group as the dependent variable. The Kolmogorov–Smirnov test suggested that the GSI was not normally distributed; however, we selected parametric tests for data analysis for the following two reasons: (1) the Kolmogorov–Smirnov test results flux and are not always reliable as the sample size varies, especially with a large sample size [56]; (2) a normal distribution of GSI was assumed in the present study, based on the histogram, the normal Q-Q plot, and the values for skewness and kurtosis [57]. Therefore, we summarized the GSI using the mean and standard error (SE). Before performing the statistical analysis, the data was preprocessed. We first discarded those values that were more than or less than 2.5 standard deviations from each participant’s mean as outliers. Then, a one-way analysis of variance (ANOVA) was applied to examine the overall difference in GIS among the different groups. When a statistically significant result was detected for the overall difference using an ANOVA, a post hoc test was performed using Fisher’s least significant difference (LSD) to investigate which group differed from the others in terms of GSI. For all statistical analyses in the current study, a *p*-value of 0.05 was considered to be the statistical significance threshold.

## 4. Results

### 4.1. Differences between the Native Speakers’ Group and EFL Groups

The participants’ grammatical sensitivity index (GSI) values were computed based on the RT difference derived from the mean RT of ungrammatical items, minus the mean RT of the grammatical items. Based on the pre-designed criterion for eliminating outliers, we excluded 105 items from the NS group, 132 items from the CS group, and 158 from the SS group. The mean GSI of the native-speaker group was 125.8 ± 25.8; for the Chinese EFL learner group, it was 40.4 ± 11.4, and for the Spanish EFL learner group, it was 58.9 ± 11.7.

The results of the ANOVA indicated a significant difference among the three groups in terms of mean GSI (F *=* 5.630, *p =* 0.004). The post hoc tests using the LSD test further suggested that the NS group had a mean GSI of 125.8, which was significantly higher compared to that in the CS group (GSI *=* 40.4, *t =* 3.04, *p =* 0.001) and SS group (GSI *=* 58.9, *t =* 2.55, *p =* 0.008) indicating that both the Spanish and Chinese advanced EFL learners did not acquire a general implicit knowledge level that was equivalent to the native speakers’ level.

However, the difference between the CS and SS groups in terms of the mean GSI was not statistically significant (*t =* −1.13, *p =* 0.272). The results are depicted in Table 2, illustrating that Spanish and Chinese learners of English do not show a significant difference in their overall level of implicit language knowledge acquisition.

### 4.2. GSI for Different Types of Morphosyntactic Errors

**Figure 3:**
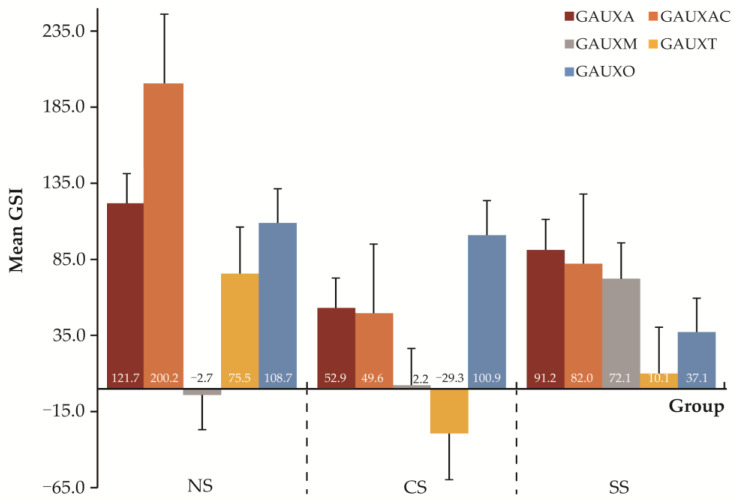
Mean GSI for each morphosyntactic error type.

For the five types of error, the results of the ANOVA for each type of error among the three groups are as follows: GAUXA (F *=* 1.19, *p =* 0.304), GAUXC (F *=* 4.24, *p =* 0.015), GAUXM (F *=* 1.506, *p =* 0.224), GAUXT (F *=* 2.691, *p =* 0.069), and GAUXO (F *=* 1.367, *p =* 0.256), suggesting a significant difference among the three groups in terms of GAUXC, that is, the choice of auxiliary. However, the mean GSI scores of the NS group are numerically higher than those of the SS group, followed by the CS group in all types of errors, except for the GAUXT items (see Figure 3). The post hoc test result with the LSD test is shown in Table 3, indicating a significant difference in terms of GAUXC between the Chinese group and the native group (*p =* 0.004) and between the Spanish group and the native group (*p =* 0.021). Meanwhile, the Chinese group showed significantly lower sensitivity compared to the native group (*p =* 0.028) in terms of GAUXT when detecting the morphological inflection related to verb tense in forming questions, while the Spanish group did not show significant differences compared to the native group (*p =* 0.163).

## 5. Discussion

The present study analyzed the GSI, as represented in the RT for ungrammatical and grammatical English questions in a WMT experiment regarding Spanish and Chinese EFL groups. To be specific, the study investigated: (1) the GSI difference between the native-speaking group (NS) and two EFL groups (CS, SS), exploring the degree of implicit knowledge of English questions acquired by advanced EFL learners; (2) the statistical results between advanced EFL learners and the NS group with respect to the morphosyntactic inflections involved in English question formation, revealing whether morphosyntactic inflections may impede EFL learners’ implicit acquisition regarding English questions.

### 5.1. Implicit Knowledge Acquisition for Advanced EFL Learners

As we hypothesized, we found a significant difference in GSI between the NS group and the two EFL groups, revealing that advanced EFL learners did not attain a general level of implicit knowledge in English questions that is equivalent to that of native speakers. As summarized in Section 2, previous research studied EFL learners’ acquisition of English questions at different levels, including Spanish learners of English, the topic of subject–verb inversion [25], a corpus analysis of child learners of English from various L1 in acquiring *wh*-questions [26], Indian learners of English [58], Cantonese learners of English [28], and Spanish and Chinese learners’ production of questions [27,48]. The studies found that learners had problems with different morphosyntactic inflections, such as subject–verb inversion or subject–verb agreement. However, no systematic investigation had as yet been conducted to explore whether advanced EFL learners can reach a native-like proficiency level with respect to their acquisition of implicit knowledge. By studying a series of potential grammatical errors, including word order, auxiliary choice, tense morphology, subject–verb agreement, and auxiliary and verb morphology, the current study found that proficiency-matched advanced EFL learners from two completely different EFL contexts consistently and uniformly showed that they were not able to acquire native-like implicit knowledge in terms of English questions. Overall, this finding contributes an additional piece of evidence to the preceding studies, supporting their results on the ultimate attainment of native-like implicit knowledge, showing that it is enormously difficult for L2/FL learners to acquire a native-like knowledge of morphosyntactic features, despite this being theoretically possible.

The present study also revealed that the two advanced EFL groups of Chinese and Spanish learners showed a similar overall level of implicit knowledge in terms of English questions. Similar conclusions were drawn on the issue in previous studies. Hopp’s [36] study on the acquisition of L2 inflection showed that it is possible for adult L2 learners to acquire native-like morphosyntactic inflection, such as L2 subject–verb agreement or case inflection; however, L1 transfer is an important constraint for L2 learners in acquiring native-equivalent accuracy of these morphosyntactic inflections. The ultimate attainment of L2 inflectional morphology is extraordinarily difficult for learners whose L1 does not have a congruent morphology, even though acquisition may be possible [39]. Even after L2 learners may have obtained high-level proficiency, they are still confounded by grammatical inflection, with morphological problems such as omission or commission in L2 production [24].

### 5.2. Major Challenges in EFL Learners’ Implicit Knowledge Acquisition of English Questions

Since we found that the two advanced EFL learner groups did not acquire a native-like level of implicit knowledge of English questions, we further explored the experimental data on five different types of errors, in order to ascertain what might constitute the specific difficulties impeding EFL learners from progressing to a native-like level of implicit knowledge. The statistical results in Table 3 revealed that neither group of participants exhibited a significant difference in most of the five types of potential errors, except in the case of GAUXC for Spanish learners, and GAUXC and GAUXT for Chinese learners.

We can relate these specific morphosyntactic problems to Jiang et al.’s [39] morphological congruency hypothesis and can investigate the three predictions that we made regarding the acquisition of morphosyntactic features in English questions by each group of learners. Advanced Spanish EFL learners encountered great challenges in detecting the GAUXC type of error, which indicated that they did not acquire implicit knowledge of the choice of auxiliary in English questions. This finding is in agreement with prediction 1 since auxiliary selection is not found in their L1, Spanish. In Spanish, no *do*-support auxiliary is required to form questions, even if the language does have auxiliaries in other contexts. Advanced EFL learners may be able to use auxiliaries on specific occasions, when they are consciously paying attention to the language forms, but the participants did not detect the grammatical violations when their focus had been directed toward key-pressing and sentence meaning in the experiment, which indicates that no implicit knowledge was available for them to recognize the error subconsciously.

As for the other four types of morphosyntactic errors, GAUXA, GAUXO, GAUXT, and GAUXM, which involve morphological inflections on number, person, and tense, Spanish EFL learners showed a high degree of implicit knowledge, as this first language displays a complex morphosyntactic system for person, number, tense, mood, and aspect features in verbs [59]. This supports the third prediction that Spanish EFL learners performed better on morphosyntactic features that were congruent with morphological inflections in Spanish.

With respect to Chinese, this language has neither auxiliaries nor morphological verb inflections of person, number, or tense (as seen in examples (1c) and (2c), above); thus, no morphological congruency exists at all between Chinese and English questions. Therefore, most Chinese EFL learners rely heavily on the grammatical knowledge they have learned at different stages, progressing to high proficiency by means of practice and communication, which facilitate their language acquisition. Although advanced Chinese EFL learners showed similar levels of implicit knowledge to their Spanish counterparts, they were confronted with more challenges in recognizing GAUXC and GAUXT errors, which means that they did not acquire implicit knowledge in either the choice of auxiliary or in verbal tense morphology in English questions. This finding is in line with the prediction that Spanish EFL learners would outperform Chinese EFL learners in acquiring morphosyntactic features in English question formation because of Spanish’s congruent morphological inflections in verbal phrases.

Our findings showed similarities and differences between advanced Chinese and Spanish EFL groups in the acquisition of the implicit knowledge of morphosyntactic features in English questions. The experimental results revealed that the degree of implicit knowledge acquisition of English questions is similar for EFL learners from two different L1 backgrounds when they are at an equivalent proficiency level. However, the two groups encountered different challenges in the acquisition of implicit knowledge regarding different types of morphosyntactic features, due to the differences in L1–L2 morphosyntactic congruity. As shown in previous studies [2,14,30,60,61], the L2 linguistic context for L2 acquisition, such as the natural communicative context or L2 country residential experience [14], affected the acquisition of implicit knowledge by L2 learners. The chi-square test results shown in Table 1 indicate that these two groups of EFL learners are at an equivalent level of proficiency and they are both from EFL contexts; however, their different learning backgrounds, such as the year of the onset of L2 learning, residential experience in target language-speaking countries, and the number of years of taking formal English classes, may also partly explain Spanish and Chinese EFL learners’ different challenges. Investigating language-learning contexts within the framework of social-cultural theory falls outside the scope of this study but certainly deserves further research.

### 5.3. Limitations

The present study was conducted within an online environment. There are many advantages of using the online experimental mode. For example, recruited participants can be drawn from different areas of the same L1 background, which may be more representative than targeting participants from a specific language class. However, the drawbacks of the present study cannot be ignored. Although we explicitly stated that participants should have a quiet and undisturbed experimental environment, quality equipment in terms of audio and recorder toolsets, a stable internet connection, and be in a peaceful state, we could not control for other potential factors that may have disturbed them, such as noise, interactions with co-inhabitants, and other distractions affecting their concentration. A pure experimental environment, such as that in a laboratory, would be optimal for the stricter control of disrupting factors. Moreover, we need to acknowledge that L2 learners are more tolerant of errors; however, the present study did not introduce acceptability judgment tasks to the model to investigate our participants’ degree of tolerance of the different types of morphosyntactic errors, which is worthy of exploration in future studies.

The hypotheses and experiments were well-grounded in previous studies; however, the sample size of this study is relatively small. In order to magnify the significance and explanatory power of the study, future studies may collect a larger sample from different proficiency levels and L1 backgrounds for more robust hypothesis support.

## 6. Conclusions

In the current study, we adopted the word monitoring task, to measure the degree of implicit knowledge acquisition of English questions for advanced EFL learners from two different L1 backgrounds and explore the potential effect of L1 morphological influence on their language acquisition process. The grammatical sensitivity index comparing participants’ RTs to (un)grammatical sentences was analyzed, and an in-depth exploration was performed with a fine-grained classification of the five different types of grammatical problems, based on the different aspects of the morphosyntactic inflection of English questions. The L1 morphosyntactic features of these questions were discussed to reveal the inhibiting factors for implicit knowledge acquisition for different advanced EFL learners. To sum up, this study revealed that: (1) it is difficult for advanced EFL learners to acquire implicit language knowledge that is equivalent to that of native speakers, despite their having acquired a high level of language knowledge and achieved high English proficiency; (2) EFL learners’ different L1 backgrounds do not affect their overall implicit knowledge acquisition; (3) EFL learners encounter distinct difficulties in acquiring implicit knowledge of morphosyntactic inflection in the context of questions, due to L1–L2 incongruency.

Future studies involving EFL learners at different proficiency levels, including beginner, medium, and advanced levels, who are drawn from different L1 backgrounds, may ideally uncover the trajectory of how the influence of the L1 evolves in the L2 acquisition process. In addition, studies investigating the language learning context within the framework of social-cultural theory may be of great significance to improving our understanding of EFL learners’ acquisition of implicit knowledge in their unique language contexts.

## Figures and Tables

**Table 1:** Demographic information for all participants.

Variables	NS Group (%)		CS Group (%)	SS Group (%)
Age		*χ^2^ =* 8.385, *p =* 0.078		
18–20	1 (8.3)		1 (3.1)	5 (13.5)
21–30	8 (66.7)		30 (93.8)	25 (67.6)
31–40	3 (25.0)		1 (3.1)	7 (18.9)
Gender		*χ^2^ =* 4.096, *p =* 0.129		
Male	4 (33.3)		10 (31.2)	20 (54.1)
Female	8 (66.7)		22 (68.8)	17 (45.9)
Education		*χ^2^ =* 6.273, *p =* 0.180		
Secondary school	1 (8.3)		3 (9.3)	4 (10.8)
Undergraduate	8 (66.7)		12 (37.5)	10 (27.0)
Graduate	3 (25)		17 (53.2)	23 (62.2)
Starting year		*χ^2^ =* 20.598, *p* < 0.001		
Kindergarten	NA		0 (0.0)	7 (18.9)
Primary school	NA		25 (78.1)	24 (64.9)
Junior secondary school	NA		7 (21.9)	4 (10.8)
University	NA		0 (0.0)	2 (5.4)
Residence in an English-speaking country		*χ^2^ =* 3.824, *p =* 0.027		
Yes	NA		0 (0.0)	6 (16.2)
No	NA		32 (100.0)	31 (83.8)
Years of taking English class	*t =* 6.507, *p* < 0.001			
	NA		12.66 ± 2.48	11.57 ± 3.97

NS, Native speaker group; CS, Chinese-speaking group; SS, Spanish-speaking group.

**Table 2:** Results of between-group post hoc analysis with an LSD test.

Comparison	MD	SE	*t*	*p*	95% CI	
					Lower Limit	Upper Limit
NS vs. CS	85.469	25.481	3.406	0.001	35.494	135.438
NS vs. SS	66.900	25.077	2.545	0.008	17.720	116.081
CS vs. SS	−18.565	16.910	−1.126	0.272	−51.726	14.595

NS, native speaker; CS, Chinese speaker; SS, Spanish speaker; MD, mean difference; SE, standard error; CI, confidence interval.

**Table 3:** Results of the post hoc comparisons according to error types, with the LSD test.

Error Type	Comparison	MD	SE	*p*	95% CI	
					Lower Limit	Upper Limit
GAUXA	NS vs. CS	68.81086	49.00933	0.161	−27.5401	165.1619
	NS vs. SS	30.48046	48.20425	0.528	−64.2878	125.2487
	CS vs. SS	−38.33040	34.16390	0.263	−105.4957	28.8349
GAUXC	NS vs. CS	150.61708	51.76794	0.004 *	48.9547	252.2795
	NS vs. SS	118.18102	50.89790	0.021 *	18.2272	218.1348
	CS vs. SS	−32.43606	35.13534	0.356	−101.4352	36.5631
GAUXM	NS vs. CS	−4.94959	67.90427	0.942	−138.8707	128.9715
	NS vs. SS	−74.77882	66.85778	0.265	−206.6360	57.0784
	CS vs. SS	−69.82922	43.57119	0.111	−155.7605	16.1021
GAUXT	NS vs. CS	104.88754	47.60696	0.028 *	11.2957	198.4794
	NS vs. SS	65.43957	46.84237	0.163	−26.6491	157.5283
	CS vs. SS	−39.44797	28.18437	0.162	−94.8564	15.9605
GAUXO	NS vs. CS	7.72514	62.69404	0.902	−115.5158	130.9661
	NS vs. SS	71.59009	61.91893	0.248	−50.1272	193.3074
	CS vs. SS	63.86496	42.72014	0.136	−20.1123	147.8422

NS, native speaker; CS, Chinese speaker; SS, Spanish speaker; MD, mean difference; SE, standard error; CI, confidence interval; * statistical significance.

## Data Availability

The data presented in this study are available on request from the corresponding author.
